# What are the experiences of team members involved in root cause analysis? A qualitative study

**DOI:** 10.1186/s12913-023-10164-9

**Published:** 2023-10-25

**Authors:** Ruth Willis, Tracie Jones, Jo Hoiles, Peter D. Hibbert, Timothy J. Schultz

**Affiliations:** 1 Clinical Governance Unit, Southern Adelaide Local Health Network, Adelaide, South Australia Australia; 2 Australian Institute of Health Innovation, Macquarie University, North Ryde, New South Wales Australia; 3 IIMPACT in Health, Allied Health and Human Performance, University of South Australia, Adelaide, South Australia Australia; 4 Flinders Health and Medical Research Institute, Flinders University, GPO Box 2100, Adelaide, SA 5001 Australia

**Keywords:** Root cause analysis, Incident investigation, Focus group, Legislative privilege, Clinical governance

## Abstract

**Background:**

Conducting root cause analysis (RCA) is complex and challenging. The aim of this study was to better understand the experiences of RCA team members and how they value their involvement in the RCA to inform future recruitment, conduct and implementation of RCA findings into clinical practice.

**Methods:**

The study was set in a health network in Adelaide, South Australia. A qualitative exploratory descriptive approach was undertaken to provide an in-depth understanding of team member’s experience in participating in an RCA. Eight of 27 RCA team members who conducted RCAs in the preceding 3-year period were included in one of three semi-structured focus groups. Thematic analysis was used to synthesise the transcribed data into themes.

**Results:**

We derived four major themes: Experiences and perceptions of the RCA team, Limitations of RCA recommendations, Facilitators and barriers to conducting an RCA, and Supporting colleagues involved in the adverse event. Participants’ mixed experience of RCAs ranged from enjoyment and the perception of worth and value to concerns about workload and lack of impact. Legislative privilege protecting RCAs from disclosure was both a facilitator and a barrier. Concern and a desire to better support their colleagues was widely reported.

**Conclusions:**

Clinicians perceived value in reviewing significant adverse events. Improvements can be made in sharing learnings to make effective improvements in health care. We have proposed a process to better support interviewees and strengthen post interview follow up.

**Supplementary Information:**

The online version contains supplementary material available at 10.1186/s12913-023-10164-9.

## Introduction

Investigation of adverse events is an essential component to patient safety and quality improvement in healthcare. Derived from manufacturing engineering, root cause analysis (RCA) is one of a number of investigation methods now widely used in healthcare [1, 2]. RCA seeks to obtain an in-depth understanding of system safety issues and to facilitate improvements by implementing recommendations [3].

While there are more than 40 RCA techniques used in healthcare, all versions use a structured process for creating chronological maps, undertaking interviews and analysing other data, and developing cause-effect diagrams and recommendations [4, 5]. A small multidisciplinary team is appointed and utilises analytical and problem solving techniques to determine causation and subsequent recommendations for improvement, which, if implemented should minimise the risk of recurrence [6].

Criticism of RCA has focused on its equivocal results in improving patient safety [7–9], its flawed focus on a “single root cause based on a simple linear narrative that displaces more complex, and potentially fruitful accounts of multiple interacting contributions to how events really unfold” (p. 417) [9] and challenges in translating RCA methods into practice [10–12]. Weak recommendations, a focus on active failures over latent organisational issues, lack of generalisability, and poor quality of RCAs are also routinely reported [4, 9, 11, 13]. Consequently, some organisations are moving towards more flexible patient safety investigation methods [14, 15] and/or refined RCA approaches [16]. Nevertheless, despite criticisms, RCAs are embedded into policy and practice in many health systems across the world and will continue to play an important role in making healthcare safer if it becomes better at delivering benefits [9]. An understanding of the issues encountered during RCAs can be used to inform changes in how they are conducted.

The RCA process is complex and challenging and there are numerous barriers to its use in practice [9, 12, 17, 18]. A procedure-oriented approach in RCAs can lead to the mishandling of emotions and blame, and have flow-on impacts on the quality of findings [18]. Interpersonal issues are especially relevant for RCA team members charged with interviewing their colleagues, with concerns about “*how to break the news to colleagues that this RCA is under way; how to make sure questions are asked in ways that do not upset people…and how to rescue relationships with the interviewees in case the latter become defensive or anxious*” (p. 1608) [19]. However, the experiences of RCA team members (i.e. those not directly involved in incident, but providing clinical expertise) has been little studied.

The aim of this study is to better understand the experiences of RCA team members and how they value their involvement in the RCA. These findings will be used to potentially inform future recruitment, conduct and implementation of RCA findings into clinical practice.

## Methods

### Study design

This study used a qualitative exploratory descriptive approach to provide an in-depth understanding of team member’s experience in participating in an RCA. Focus groups were selected to explore social dynamics and interactions between participants increasing the depth of the enquiry, highlighting similarities and differences between participants, and understanding their perspectives and experiences [20, 21]. The study was conducted and is reported according to the COnsolidated criteria for REporting Qualitative research Checklist [22].

### Setting

The local health network in this study is based in Adelaide, South Australia. It has adopted RCAs (with legislative privilege (Part 8 protection) [23] that protects all detailed RCA reports from disclosure to clinicians and managers and to legal proceedings) to review significant adverse incidents and ascertain the ‘what, how and why’ of identified patient safety incidents’. Legislated privilege is State-based legislation that is in place across most Australian States [24, 25]. RCAs are commissioned by the organisational adverse events committee; there were 7 RCAs completed in 2019 and 3 in 2020. The RCA process is managed by the health network’s Clinical Governance Unit, which provides leadership and team support. The lead investigator will have completed formal RCA training, other members of the team are typically clinical staff with expertise in the area being investigated, and engagement of a consumer representative is sought where appropriate. The RCA process involves team formation, meeting to review the patient journey and determine interview requirements. Following interviews, the team reports back to the adverse events committee, including a causation statement (if determined) and recommendations. Recommendations are then refined prior to sending to the allocated person for action.

### Recruitment

Two emails over a 3 week period were sent to all 26 clinicians and one consumer who were team members in any of the ten completed RCAs in the preceding 2-year period. One participant was no longer working within the network and was excluded from the study. Ten people, comprising nine staff members (including six medical, two nursing and one pharmacy staff) and a consumer representative agreed to be interviewed; however, the involvement of two medical staff was unable to be scheduled successfully.

### Data collection

Data was collected in three semi-structured focus group interviews conducted in face-to-face (n = 1) and online (MS Teams) format (n = 1) [26], and a blended session comprising both face-to-face and online participants. Two researchers (TS, an experienced health services researcher who did not previously know the interviewees, and either of RW or TJ, trained facilitators for RCAs as a co-interviewer) conducted the three focus groups using a topic guide (see supplementary file), which was developed by RW, TJ and TS based on relevant literature and reviewed by an expert in the conduct of RCAs (PH) and consumer representative (JH). The co-interviewer provided insight into local context in RCA conduct; to promote full and frank discussion the co-interviewer was selected on the basis of not being involved in RCAs that were discussed. Face-to-face interviews were conducted in University-managed space within the health network in September 2021. Focus group discussions lasting 45–60 min were recorded and transcribed.

### Data analysis

We conducted a thematic analysis of the transcribed data using an iterative coding process [27]. Researcher RW inductively derived first cycle in vivo codes from participants’ own words by RW, then clustered similar codes together in second cycle pattern codes [27]. This analysis was conducted manually in MS Word and then reviewed by TS and TJ with further discussion of coding discrepancies. The three analysts then collected similar pattern codes into higher order sub-themes and themes that described the participants’ experience of participating in RCA. The sub-themes and themes were subsequently reviewed and verified by PH and JH. Participant checking of data analysis was not conducted.

## Findings

### RCA and interview participant characteristics

There were interview participants from five of ten completed RCAs (Table 1). Each of the completed RCAs had 4–5 team members, comprising 2–3 clinicians and 1–2 clinical governance staff. Five RCAs only included doctors as clinicians. Two RCAs included one safety and quality staff, and one RCA included a consumer.

**Table 1:** Breakdown of team member participation in 10 RCAs completed from 2019–2021;

	Clinical	Consumer	Governance	Safety & Quality	Total
**RCA #**					
1	3	0	1	0	4
2*	3	0	1	0	4
3	2	0	2	0	4
4*	3	0	1	1	5
5*	3	0	1	0	4
6*	2	1	1	0	4
7	3	0	2	0	5
8	3	0	1	0	4
9*	3	0	1	1	5
10*	3	0	2	0	5
Total	28^$^	1	13	2	44

* at least one interview participant was a team member. ^$^Two clinical RCA team members were involved in two RCAs

Of eight participants, there were four doctors (three consultants, one senior registrar), two nurses, one allied health and one consumer. Three interviewees (doctors) had been involved in multiple RCAs over their careers.

### Themes

We derived four major themes, each comprising two to four sub-themes (Table 2).

**Table 2:** Analytical themes and sub-themes

	Themes		Sub-theme
1	Experiences and perceptions of the RCA team	1.1	Mixed experiences
		1.2	The value of RCAs
		1.3	Responsibility to contribute to patient safety
2	Limitations of RCA recommendations	2.1	Challenges in implementing recommendations
		2.2	Sharing recommendations more broadly
		2.3	Feeding back implementation of recommendations
3	Facilitators and barriers to conducting an RCA	3.1	Facilitation by training, organisational support, trust and virtual meetings
		3.2	Impeded by lack of proper preparation and protected time, short timeframes and moving jobs
		3.3	Protection from discovery as a facilitator and barrier
4	Supporting colleagues involved in the adverse event	4.1	Empathy for colleagues
		4.2	Structured organisational support and acknowledgement

#### Theme 1 – experiences and perceptions of the RCA team

Participants described a mix of positive and negative experiences from their role on the RCA team: *“I had a very good positive experience…” (P2, FG3)* and *“I have enjoyed being part of the RCA process” (P2, FG3)* and *“I was very happy and well prepared, and it was managed extremely well by Clinical Governance” (P2, FG3).* One participant described the process as inconvenient but worthwhile “*Overall it was worthwhile, but just quite…just hard when you’re so time poor and it’s very hard to find the time to get away and attend the meetings*” (P3, FG3). However, one participant did not enjoy the experience.

*“Looking back, I didn’t feel very comfortable about the whole process and that was probably partly because I didn’t understand really well or I had never done it before, so I didn’t know what to expect, it wasn’t something I enjoyed or felt very comfortable doing in all honesty” (P1, FG 3).*

Participants generally agreed the RCA review process was worthwhile and of value. On the whole, team members learnt about RCAs and how errors occur through the RCA process, and believed the investigation and subsequent recommendations would prevent a similar incident recurring.

*“I think it is a very valuable thing, cause that’s how you get to find out what are the issues, what are the problems in the process in the interventions we can implement that will improve the patient care or the patient journey in future, so I still think it’s really important to keep doing this” (P 1, FG 2).*

A number of participants described the RCA process as learning opportunities facilitating reflection on their practice: “*It feels quite robust and … there’s always a point to it. I usually come away having learned something from it*” (P3 FG3). However, a participant was ambivalent about the impact of RCA stating “*I don’t know if it led to significant change” (P3, FG 3).*

RCA team members felt responsible to contribute: “*I work in a public hospital, so it is my responsibility to have to contribute to change the system for benefit of the hospital” (P 2, FG 1).* Staff reconciled the additional workload from the RCA as part of their work duties “…*it was long and drawn out and is additional workload, but I felt it’s my responsibility to understand and be supportive*” (P 2, FG 3).

#### Theme 2 - limitations of RCA recommendations

While most interviewees acknowledged benefits from RCAs, a number of limitations with the derived recommendations were noted. ‘Gold standard’ recommendations were difficult to implement in practice:

*“There were lessons learned and it highlighted significant issue with communications and hopefully will have a positive outcome for communication. The recommendations that we implement are gold standard … but reality is there are times when the ideal can’t be met for practical reasons, and that is what is disappointing.” (P2, FG 3)*.

Many adverse events investigated using the RCA methodology are common problems, sometimes despite being addressed in previous RCA recommendations. Many participants felt that the learnings from RCAs should be shared more broadly given the amount of resources invested in conducting the RCA.

*“I do get a sense that sometimes we sort of feel like we’re in a bit of a revolving wheel and we see the same thing over and over, and sometimes it might be that we do a review for something that happens, and the very same thing might be happening somewhere else” (P 3, FG 3).*

*“It would be useful to discuss if recommendations should be distributed throughout the state, or just in this network” (P 2, FG 1).*

*“I think there is significant opportunity for us to be able to share the learnings from RCAs across the local health networks, but I feel it is very much a closed shop and yet there’s a number of recommendations that I have been aware of through work with different colleagues” (P 1, FG 2).*

Participants sought feedback about implementation of recommendations:

*“In a perfect world, if we received say an update six months later, about [how] the recommendations were actually implemented” (P 3, FG 3).*

#### Theme 3 – facilitators and barriers to conducting an RCA

Participants described a number of facilitators and barriers to conducting RCAs. The facilitators included undergoing training “*Training was clear and useful…the person running the course was a great help*” (P1, FG1) and support provided by the Clinical Governance Unit:

“*They were very good at keeping you up to date and organizing the meetings… I just get to ask the interesting questions and kind of take the cognitive load on about what does that mean*” (P2 FG3).

Putting their trust in the process was also an important facilitator: “*Not everything is under my sphere of influence and so you know there comes a period of trust where I have to trust that someone else doing that [recommendations] as well*” (P3 FG3). Another participant stated *“So the assumption is that they [consultant] would take carriage of implementing with their work group recommendations*” (P2 FG2). Virtual meetings helped to facilitate involvement of staff across different sites.

Being properly prepared was a barrier:

*“The first one, I didn’t know the background really of RCAs. It was hard the first time; I was surprised re the time commitment and wasn’t aware of the need to go and interview and then the meetings afterwards*.*” (P2, FG 1).*

The short timeframe available was a barrier: “*but we had a very short time frame to complete it in. It was quite urgent. So it had to be sort of rushed through quite quickly”* (P1, FG 3). Moving jobs was mentioned by two participants as a barrier, and one of the more junior staff did not expect much organisational support to allow them to participate: “*I mean, you never really have time allocated for these kinds of things. You have to work around the job …. I didn’t really discuss it with my line manager*” (P3 FG3).

Legislative privilege means that detailed findings are protected from discovery not only media or legal proceedings but to clinicians and managers [23]. The legislative privilege was both a facilitator and a barrier to RCAs. Many participants believed the legislative privilege provided the interviewees with the freedom and willingness to discuss the clinical incident freely:

*“…you ‘don’t get open and honest disclosure unless you are protected, especially the more informed professional groups” (P 2, FG 3).*

*“I think Part 8 protection is very important for actually getting to the source of things cause it meant people are much more willing to open up and discuss things openly” (P3, FG 2).*

There were, however, mixed opinions on the lack of transparency in RCAs:

*“…some individuals are quite uncomfortable about the Part 8 process - it should be something that is completely in the public domain” (P2, FG 2).*

*“You know, can we be laid open to the criticism that you’re closing ranks and protecting your own? And as opposed to actually truly telling the public what may or may not have gone on?”. (P2, FG3)*

There were also comments indicating a lack of clarity about the legislative privilege, and why it exists. Although details of RCA investigations cannot be shared, this poses an issue when attempting to ‘share’ learnings from investigations.

One participant neatly summarised the complexity of legislative privilege in RCAs: “*it’s very hard to actually produce something which can be usefully applied to change practice without providing details, but I think the confidentiality and protection is really important part of the process because it means that people can reflect honestly on what happened rather than necessarily being overly protective. So I think it is very hard to get the balance right*” (P1, FG3).

#### Theme 4 - supporting colleagues involved in the adverse event

Participants expressed their concern for staff being interviewed and noted the emotional impact of the RCA on staff: *“…it (the interview) was quite emotional, and some staff broke down in tears, so it was actually a very harrowing experience to hear what happened to an individual patient” (P 2, FG2).* The impact was compounded by a lack of full understanding: *“I completely agree some of the clinical staff that I have interviewed have sleepless nights leading up and still not understanding completely the finer details of the process” (P 1, FG 2).* The resulting concern by team members contributed to a feeling of unease about the interviews:

*“I had some trepidation about interviewing colleagues, a little concerning as conducting an RCA they were involved in and perhaps the perception they are being challenged in their performance” (P 2, FG 1).*

This concern led to a desire to support RCA interviewees as individuals and to suggest that more organisational support be offered. One RCA team member said “*I felt it’s my responsibility to understand and be very supportive. So I was present during the interview of all my nurses*” (P2 FG2). Organisational support could be provided through a third party not included in the RCA or their clinical team:

*“Identify somebody outside of the RCA group who is supporting staff through these, sitting down explaining the process, meet with them both leading up to the RCA and afterwards so they’re feeling like that they’re not either having to go to their manager. They have someone neutral that they can go to for support and understanding the process, somebody that sits outside of the risk”* (P1, FG 1).

Interviewees could also be provided with more organisational recognition and acknowledgment, recognizing that some staff may prefer to remain anonymous:

*“We should acknowledge people more for their bravery to participate, to take the fear out of it. I think some correspondence from someone senior saying this is very valuable” (P1, FG 1).*

*“…a letter for those who were interviewed, thanking them for their participation. A letter can discuss what happened and recommendations, stating you have made it possible to make the changes to draw a conclusion to the particular episode” (P2, FG 1).*

Reinforcing the ‘no blame’ culture would also help to provide more support to RCA interviewees: “*But also, I think believing that when we say this isn’t about pointing the finger, this is about identifying systems errors” (P 1, FG 2).*

## Discussion

This study is important because it addresses a research gap in RCA team members’ experiences of RCAs. RCA team members reported mixed experiences of RCAs, ranging from enjoyment and the perception of worth and value to concerns about workload and lack of impact. Nevertheless, the RCA team members learnt from the process and it led them to reflect about their own practice. Similarly, it has been shown that involvement in RCAs may improve patient safety culture [28, 29]. This may be facilitated by improved teamwork and communication, and trust and openness, from bringing staff together from different disciplines [12, 29, 30]. Our study also suggests that staff willingness to undertake the responsibility of extra duties related to RCAs is important to the conduct of RCAs. This study did not identify conflicts between RCA team members and clinicians or unwillingness to participate in the investigation process [29].

These study findings are notable in light of the widely reported limitations to RCAs and implementation of its recommendations [8]. For example, recommendations are difficult to implement [31], result in little change [7, 9, 10], and are not widely shared, sustainable or connected with practice [18]. Additionally, despite a perceived need to be aggregated in order to generate system level improvements, this does not routinely occur [32], resulting “in failure to disseminate painfully acquired learning and to address deeper, institutionally engrained patient safety concerns” (p. 418) [9]. The type and strength of RCA recommendations may be classified using US Department of Veterans Affairs criteria [4, 33, 34], generally, system-based interventions such as forcing functions and automation or computerisation are more effective than person-based approaches [35].

Our participants reflected these known concerns about RCA recommendations, in particular the possible benefits from aggregation of RCA recommendations across local health networks. However, a barrier to sharing of learnings and recommendations across networks is that the legislative privilege reduces the amount of detail that can be provided in recommendations, such that “*it’s very hard to actually produce something which can be usefully applied to change practice without providing details”* (P1, FG3). The intention of legislative privilege is to encourage health service staff’s voluntary participation in RCAs and assure interviewees directly involved in the incident that the investigation is confidential and protected from discovery, for example, by the media. The legislative privilege is a balance between providing confidentiality to promote willing and robust participation, and some transparency about processes and outcomes that can permit others to learn from lessons identified by the RCA team [24]. Herein lies the trade-off at the heart of the legislative privilege – while it enhances disclosure it limits the utility of the consequent recommendations – such that it seems unlikely that sharing of reports and recommendations across networks can occur under legislative privilege. Nevertheless, some local health networks, are currently piloting the conduct of some RCAs without privilege; this may provide an opportunity to test the sharing of RCA recommendations and whether the inherent desire to protect reputation that impedes identification of organisational failures can be overcome [36]. In other jurisdictions, the final RCA report may be shared to assist implementation, while the interviews cannot be shared [14].

Participants referred to recommendations using terms such as ‘gold standard’ and ‘very strong’, which contradicts a number of retrospective studies that describe predominantly weak recommendations from RCAs [4, 17]. This disparate finding may reflect the different perspectives of those embedded in the development of recommendations. The participants trusted the process of the RCA and that implementation of the recommendations would take place outside of their spheres of influence. However, if RCA participants with intimate knowledge of recommendations were more involved in their implementation [31] then this would help to inform the implementation and keep participants informed about the implementation, thereby addressing barriers raised in this study. The literature suggests that there are few incentives to formally follow-up on the implementation of recommendations. While 45–70% of recommendations may be implemented, they may not be implemented thoroughly or may be abandoned shortly thereafter [9, 37].

Participants’ expressions of concern and desire to support their colleagues being interviewed during the RCA was a very strong theme of the focus groups. Although intended as a rational activity, the interaction of the socio-technical aspects of the RCA are emotionally fraught with anxiety, fear and shame, and concerns about judging clinical practice and being agents of ‘the bureaucracy’ [10, 18]. Guthrie emphasises the requirement to ensure psychological safety for participants and acknowledges the interview process can be intimidating and ‘scary’ for staff [38]. Accordingly, interviewers should be empathic and aware of vulnerability of the staff whose actions have potentially contributed to an adverse outcome [38]. The participants also recommended that supports should be implemented acknowledging staffs’ participation. This support could be in the form of a letter, a neutral support person or some other correspondence from a senior staff member.

### Limitations

We were only able to recruit 2–3 participants each to a total of 3 focus groups. The small number of participants per focus group may have limited the interaction between participants, and thereby the effectiveness of the method. Nevertheless, the results obtained were sufficient to provide a clear understanding of experiences across a range of clinical backgrounds. Although the findings were generally consistent, the inclusion of more focus groups and/or participants may have led to new findings. We do not claim that data saturation was reached, but note that the importance of saturation in determining sample size of qualitative studies is increasingly contested [39, 40]. As a group activity, focus group participants may be less inclined to divulge confidential, or less socially acceptable viewpoints. Additionally, some participants worked with and knew the Clinician Governance Unit-based co-interviewers, which may have impacted their responses. Finally, those who declined to participate may have had negative experiences with RCAs, which could lead to the under-representation of negative perspectives.

## Conclusion

RCA team members suggested that participating in RCAs was a worthwhile process that appropriately explored key system issues involved in adverse events. RCAs were perceived as promoting a safety culture for the organisation, however, barriers to participating in RCAs resonated with other studies. The use of legislative privilege hindered the sharing of meaningful learnings for dissemination across the wider health service. Implementing RCA recommendations in isolation, without a comprehensive understanding of the details of the incident, potentially impacts on uptake.

This study should reassure clinicians of the perceived value of RCAs in reviewing significant adverse patient incidents. Improvements can be made in the way that learnings from RCAs can be shared to make effective improvements in health care. This study builds on current literature by suggesting a process to support interviewees and strengthen the process for post-RCA interview follow up and acknowledgement of RCA participation.

### Electronic supplementary material

Below is the link to the electronic supplementary material.

Supplementary Material 1

## Data Availability

The datasets generated during and/or analysed during the current study are not publicly available as participants did not consent to this, but are available from the corresponding author on reasonable request.
