# Supplementary material for: What are the experiences of team members involved in root cause analysis? A qualitative study
Source: BMC Health Serv Res. 2023 Oct 25;23:1152. doi: 10.1186/s12913-023-10164-9 (PMC10601107; doi:10.1186/s12913-023-10164-9)
Supplement: Supplementary file 1 — Supplementary Material 1 [file 12913_2023_10164_MOESM1_ESM.docx]

**Interview Questions/guide**

*Thank you for participating in this project.*

*The aim of this study is to explore your experience as a team member participating in a Root Cause Analysis, for example, we would like to understand whether you feel there was value in your involvement in the RCA process, and your perception of how recommendations were developed and finalized, and what works in implementing RCA recommendations.*

*The findings from this study will help inform how we can best review adverse patient incidents and how best to utilize the skills of our clinicians and staff to assist in this process.*

*The intention is not to discuss the actual details of the case, which, as a Part 8 activity, are protected from disclosure, rather the intention is to discuss the RCA process.*

To help inform the analysis and the focus group discussion, it will be helpful if each participant could briefly describe:

- Their role in the RCA (team) that is being discussed today
  - How/why they were invited to the team
  - What role they fulfilled
- Their previous experience with RCAs, and patient safety initiatives, including any formal training

**Focus group discussion guide**

Based on your previous experience with RCAs, and/or other quality improvement activities, what were your expectations of the RCA experience?

- What is an RCA?
- What was your understanding of the RCA method, intentions, privilege, and how the adverse event was selected for an RCA?
- Were you concerned about being involved in the RCA, was it easy/hard to convince you to be involved? What, if any, were your pre-conceptions of the RCA?

How did you feel about doing an RCA?

- Were your expectations met, and if so, how did this happen, can you give an example of this. If not, can you explain how/why your expectations were not met, using an example?
  - If not met, do you have any other suggestions about another way to review and respond to serious adverse events?
- How important to you is it that the second part of the RCA (the full report) is protected from discovery under part 8 of the Health Care Act? Did it have any impact on your decision to participate? How did it affect others’ decisions to be interviewed as part of the RCA?

What are some words that you would use to describe the overall experience of the RCA?

*PROMPTS for the investigator*

- *feeling supported by their colleagues, managers and clinical governance unit, and hospital administration in doing the RCA?*
- *Having enough time/resources/training/support*
- *valuable use of your time?*
- *participation / input was valued?*
- *feeling heard / communication within the team?*
- *able to effect change with your recommendations*
- *involvement of consumers?*

What has happened since your RCA?

- If recommendations have been implemented successfully,
  - how has this been achieved, and why (ie on what basis) do you think the implementation is successful? (Have you seen evidence of changes in practice based on the recommendations from your review?)
- If recommendations have not been implemented successfully, or if you don’t know about the implementation of recommendations:
  - Why do you think this is the case (ie on what basis do you say the recommendations have not been implemented successfully)
  - What did you think of the strengths / weaknesses of the recommendations?
  - do you have any sense of what “happened” to the RCA recommendations?, or
  - What could have happened differently for a successful implementation?
  - How did you find the experience with the CRC when presenting the recommendations?

Can you please tell us if participating in an RCA has affected your own clinical practice?

The RCA is designed to reduce similar incidents occurring again. Is there any way that the process be modified to improve their effectiveness, and/or sustainability in reducing similar incidents?

What are your thoughts about the value of RCAs in terms of weighing up the costs and benefits?

- How do the costs and benefits impact on your decision to participate in an RCA again?
  - If you would not participate again, are there any changes to the RCA process that what would make you change your mind

Any further general comments/feedback?
